# PSYCHOLOGICAL APPROACH IN COSMETIC DERMATOLOGY FOR OPTIMUM PATIENT SATISFACTION

**DOI:** 10.4103/0019-5154.62733

**Published:** 2010

**Authors:** Mohamed Lotfy Elsaie

**Affiliations:** *From the Department of Dermatology and Cutaneous Surgery, University of Miami, FL, USA. Department of Dermatology and Venereology, NRC, Cairo. Egypt.*

**Keywords:** *Aesthetics*, *cosmetic surgery*, *psychology*

## Abstract

Knowledge and skills are required to deal with certain skin disorders and their corresponding cosmetic complaints. The field of cosmetic dermatology is growing as an overlap between the medical treatment of skin diseases and traditional cosmetology. This poses problems for dermatologists and other professionals, including regulation agencies. Dermatology should enable patients to benefit from all that is necessary for their care, whether that is surgery, drugs or cosmetics. There is no need to modify current regulations. A patient-orientated approach is advocated. The author tries to address fellow Dermatologists and those dealing with cosmetology on how to optimally maximize the outcome of their results by simple advices on how to deal with their patients.

## Introduction

As cosmetic dermatology becomes increasingly popular across all sections of the population, proper patient selection becomes even more important to the entire cosmetic process. Aesthetic dermatology is unique because, unlike any other procedure, it is initiated by the patient and not by the physician. As the patient is the initiator, cosmetic dermatologists are required to recognize the potentially complex psychological milieu of aesthetic procedures. Before proceeding to the operating room, evaluating the patient's psychological condition is essential. This can be effectively accomplished by carefully evaluating a patient's general demeanor and personality, recognizing the patient's true motive, knowing good from poor prognostic indicators and using accurate assessment technique. A cosmetic dermatologist's first impressions within an evaluation of a patient often prove most useful for gauging the suitability of a procedure. The patient's general appearance, demeanor, and behavior can also serve as indicators of a concealed psychic disturbance. Diagnostic acumen requires an awareness of subtle signs suggestive of potential problems, and the physician must practice intuitive observation of the patient at all times. Is the patient's dress provocative and alluring? Does the patient retreat from physical contact or actively avoid eye contact? How is the patient's affect and mood? Is the voice monotone or easily excitable? Cosmetologists can expect their patients to be nervous and self-conscious during consultation; however, such patients may need additional observance because nervousness may be an indicator of some emotional disturbance.[1–4]

Some overlap is inevitable among healthcare providers. In the case of aesthetic medicine/surgery, there is an overlap between plastic surgeons, dermatologists, general practitioners, nurses, dental surgeons, and other healthcare providers like ENT surgeons, head and neck surgeons on the one hand, and cosmetologists or beauticians on the other. Such overlap should be complementary rather than competitive. Over time, the pricing mechanism, difficulty of the procedures, and patient's safety factors will distribute the turf, but this can be speeded through discussion in a multi-disciplinary body like the Cosmetic Surgery Interspecialty Committee in the United Kingdom. A similar body that Singapore could consider setting up would be an Aesthetic Medicine/Surgery Interspecialty Committee. Such a body could also define the training, facilities, patient safety, patient education and empowerment for the primary care level and specialist level of practice.

Anxiety is common at the first meeting and can hamper retention, expression, and comprehension of the goals, benefits, and complications of surgery. Consider a follow-up visit for any patient with suspected emotional instability. Additionally, dermatologists are faced with inorganic complaints which may cause significant distress. Dysmorphophobia leads to cosmetic demands which may be better treated with a psychiatric approach than by cosmetic techniques.[2]

## Assessment Technique

A cosmetic dermatology patient should be comfortably seated to alleviate all tension. The patient's feet must be touching a ground to alleviate any discomfort. Many patients do feel disturbed and uneasy about seated with legs hanging up in the air and mostly due to psychological feeling of being trapped and with no self control. Head facing upright establishes a good musculoskeletal relaxation that would contribute to the patient's well being and enables him to feel comfortable. The patient must be gently approached and given time to explain and has his cosmetologist seated while describing his complaint for a better feeling of concern.

Every cosmetologist should develop an assessment technique or a preoperative screening method to review clients for emotional and psychological stability. No quick solutions or tests exist, and a method that may work well for one cosmetic dermatologist may not work well for another. Every Cosmetologist has different opinions about who is appropriate for surgery. Despite the absence of an established system, the most conservative mode is a formal psychological or psychiatric evaluation. However, this method should be reserved for patients who are psychotic or emotionally unstable. Perhaps, the most effective method of assessing a patient's psychological suitability for a procedure is simply to spend time with the patient gathering information. Cosmetologists, like any other physician, must devote significant time to an examination into the patient's family, medical, and surgical histories. Knowledge of any possible allergies, medication being taken, and smoking and alcohol use is significant for identifying specific impacts of potential complications (e.g., aspirin use, suitability of patient with significant heart disease). In addition, a thorough study of the patient should demonstrate concern for the total patient, which can possibly ease anxieties and improve communication; the patient shouldn't be merely regarded as a procedure seeking individual.

Questions aimed at assessing a patient's psychological suitability should be open-ended to allow for elaborate and informative answers. Asking open questions, such as *“How long have you wanted this change?”* rather than asking a direct question, such as *“Why do you want this done?”* may reveal much more information about issues such as the patient's motivations and psychological well-being. The open question is less threatening and offers more opportunity to elaborate on other issues. Other important questions may be, “*Do you think this technique will make any other things different or better for you*?” and “*Does anyone else want you to have this procedure*?” Furthermore, ascertaining a patient's current life situation, which is often related to the psychological condition of the patient, is also helpful. Questions regarding marital status, work type, and job satisfaction can provide further vital information to the cosmetologists when assessing the patient's overall psychological suitability.[4]

### Cosmetic Dermatology Patients: Whom to be Selected and Avoided?

The S.T.E.P technique encompasses psychological approach to your cosmetic dermatology patient.

#### S “Stress”

Identify the patient stressors and specify their importance, for example, glabellar lines or wrinkles. See those stressors and make sure they are realistic and not being exaggerated. Ensure you have the tools for dealing with them and be up to patient's expectations.

#### T “Target”

Let the patient target specify the area needed for correction. Ensure the goals are realistic and attainable otherwise explain expected outcome. Limit your focus on one target at a time.

#### E “Envision”

Ask the patient to envision hoe their perceptions, emotions and life would be different/better after the intervention. Does it sound fantasies or realistic and can you deliver?

#### P “Proactive”

If clear attainable goals and realistic expectations are present, be proactive and devise specific treatment plan for patient.

### Contraindicated and Ideal Patients

The author best recommends avoiding procedural approaches in the following patients in all aspects: (i) one with self mutiliation, (ii) major depression, (iii) troubled or agitated on the day of procedure, (iv) patients with suicidal thoughts, and finally (v) psychotics. On the other hand, an ideal candidate would be one with (i) no obvious psychopathology, (ii) clearly defined areas of dissatisfaction, (iii) realistic expectations and last but not the least (iv) a person who has motivation from or to self.

## Conclusion

The increased popularity of cosmetic procedures among virtually all demographics has compelled the identification of unsuitable candidates. A patient's psychological health is pivotal to predicting his or her satisfaction with the surgery. Emotional and psychological evaluation begins when the patient enters the cosmetologist's office. A careful assessment of the patient's general demeanor and personality type is essential. In addition to the technical excellence, perhaps the most important predictor of a successful outcome is having the right motivation for a procedure. Internally derived pressure instead of an externally derived pressure is the healthiest motivation. Proper assessment should lead to an informed opinion about a patient's psychological suitability for cosmetic procedure and the potential for a successful outcome.
